# Supplementary material for: L-Alanine Prototrophic Suppressors Emerge from L-Alanine Auxotroph through Stress-Induced Mutagenesis in Escherichia coli
Source: Microorganisms. 2021 Feb 25;9(3):472. doi: 10.3390/microorganisms9030472 (PMC7996224; doi:10.3390/microorganisms9030472)
Supplement: Supplementary file 1 [file microorganisms-09-00472-s001.pdf]

Supplementary Figures and Tables

(a)

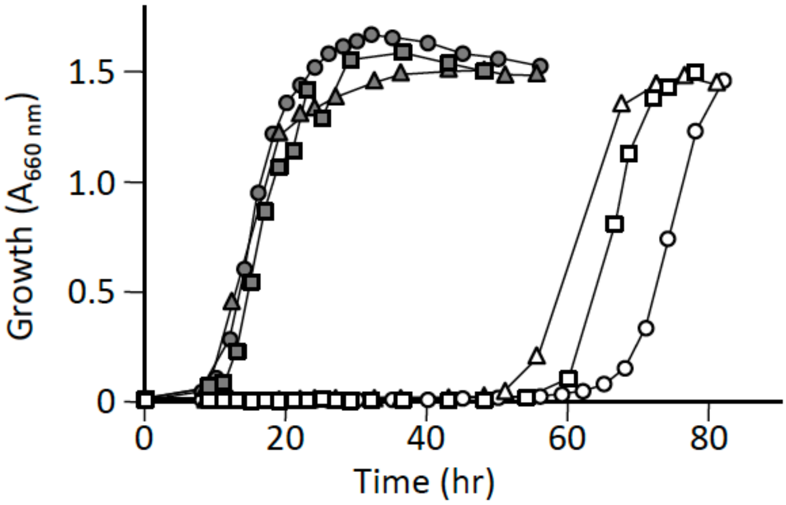

(b)

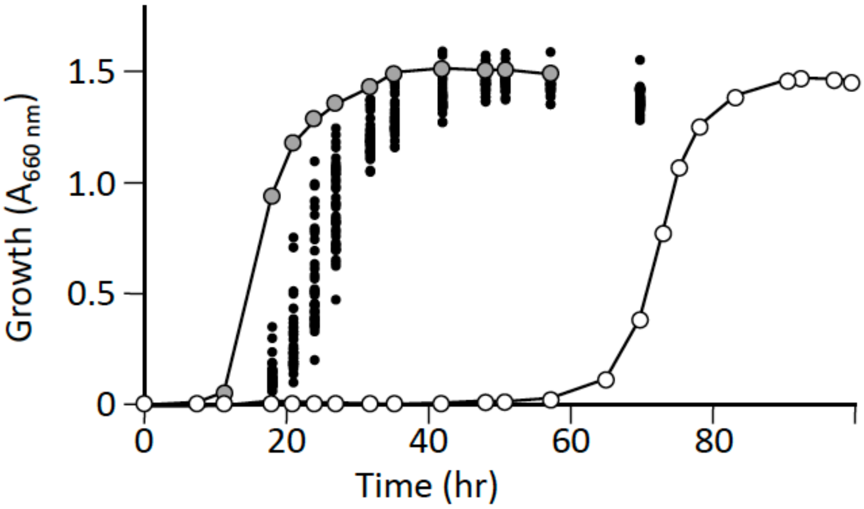

Figure S1. Emergence of L-alanine prototrophic mutants from L-alanine auxotroph HYE032. (**a**) Growth of L-alanine auxotroph HYE032 (open symbols) and its parent strain W3110 (grey symbols) in minimal liquid medium. Results of 3 independent experiments are presented. (**b**) Growth of L-alanine prototrophic suppressor mutants obtained in minimal liquid medium. When the growth of HYE032 cells reached the late-log phase in minimal liquid medium, clones were isolated on L-agar medium and each clone was purified once on the same rich medium. Subsequently, the growth of each L-alanine prototrophic mutant was determined in minimal liquid medium. Symbols: large open circles, HYE032; large grey circles, W3110; small closed circles, L-alanine prototrophic mutants.

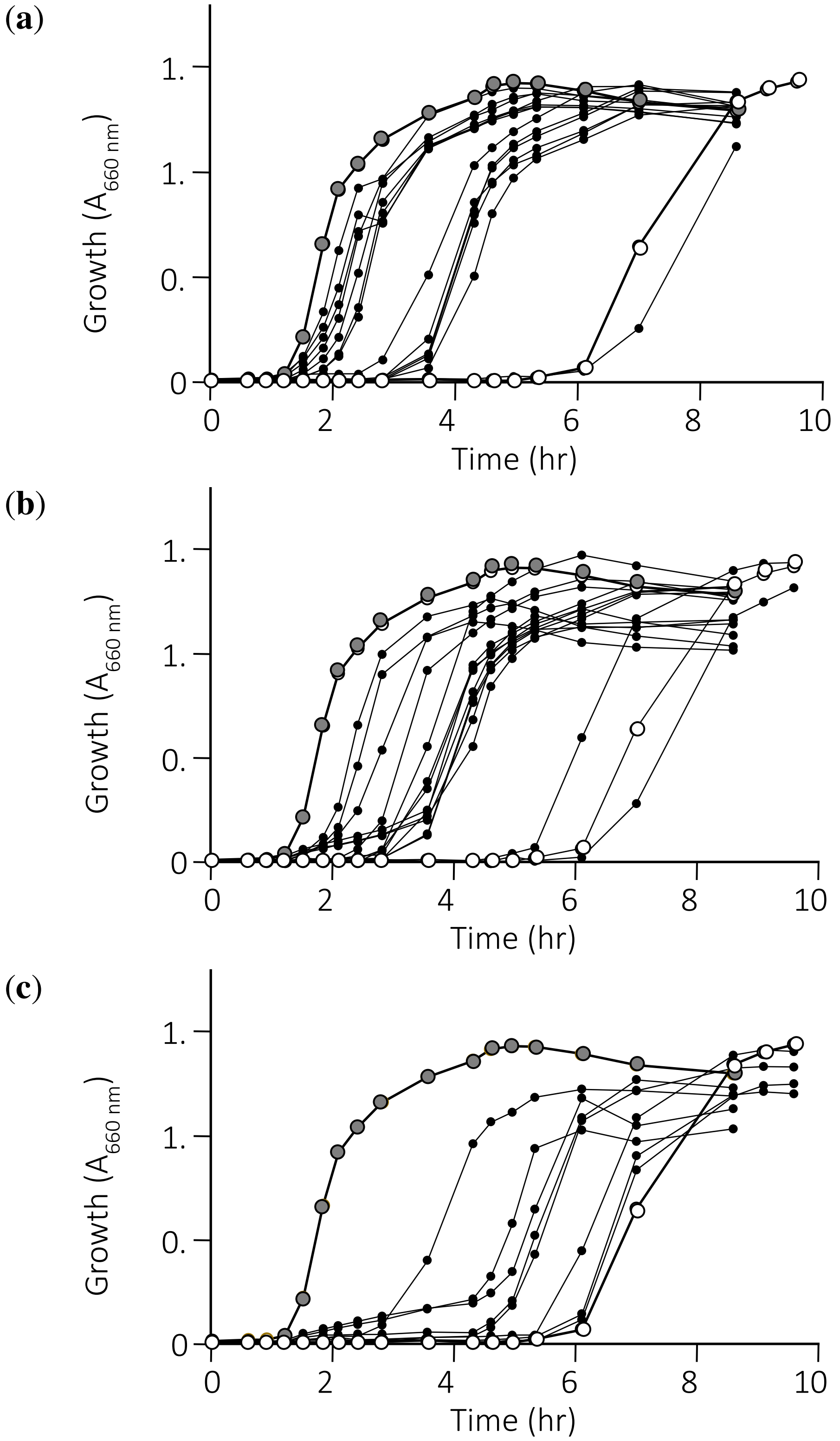

Figure S2. Growth of L-alanine prototrophic suppressor mutants obtained on minimal agar medium. L-alanine prototrophic suppressor mutants were obtained after incubation at 37°C for 2 days (**a**), 3 days (**b**), and 4 days (**c**) on minimal agar medium. Subsequently, growth of individual suppressor mutants in minimal liquid medium without L-alanine supplementation was measured. Symbols: large open circles, HYE032; large grey circles, W3110; small closed circles, L-alanine prototrophic mutants.

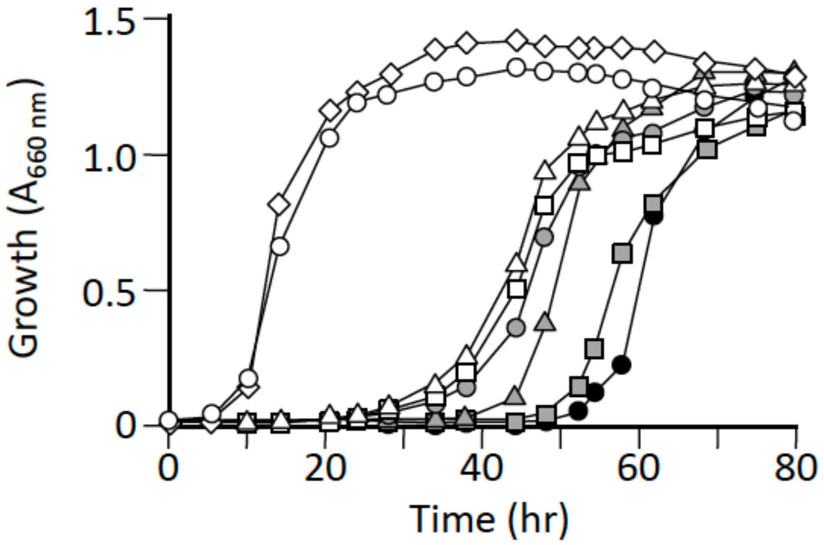

Figure S3. Effect of L-glutamic acid on growth of L-alanine auxotroph HYE032. HYE032 cells were inoculated into minimal liquid medium containing various amount of L-glutamic acid (10–2,000 μg/mL), and growth was monitored by measuring the absorbance at 660 nm. Symbols: open diamonds, W3110 with no supplementation; open circles, HYE032 with 100 μg/mL L-alanine; open triangles, HYE032 with 2,000 μg/mL L-Glutamic acid; open squares, HYE032 with 1,000 μg/mL L-Glutamic acid; grey circles, HYE032 with 500 μg/mL L-Glutamic acid; grey triangles, HYE032 with 100 μg/mL L-Glutamic acid; grey squares, HYE032 with 10 μg/mL L-Glutamic acid; closed circles, HYE032 with no supplementation. The figure shows representative results from two experiments.

Table S1. Primers used for sequencing of candidate genes.

Primer name	gene	Nucleotide sequence (5' to 3')
aceE-Rev3	*aceE*	catctAGATGTGGCCCTGGAAGTAAACC
aceE-Fwd3		CGTGATCAAAGTGATGTGGG
aceE-Fwd		TAACCACTTCTTCCGTGCACG
aceE-Fwd2		AAAAGACCTCGAACTGGGC
aceE-Rev2		TCAGCTGGATCAGTTTACCG
aceE-Fwd1		tgtctagAACAGAAGTCGTGAAGAGAGCC
aceF-Fwd	*aceF*	agtctaGACTTCAGCAAGTTTGGTG
aceF-Rev		actctagaGTAATACCCTAACCACCACC
lpd-Fwd	*lpd*	gctctagaGCAGCTGACAAAGACATCG
lpd-Rev		gttctagaAACCCATTTCGATTGCCAGG
pta-Fwd1	pta	TTCCGTGTTCTTCATGCTGC
pta-Rev1		GATGACGAGATTACTGCTGC
pta-Fwd2		TGGTTCGTGAACTGTCTCTG
pta-Rev2		AGTTTGCGACGATCTCTTCC
pta-Fwd3		ATCAGCTGACTGAACTTGCG
pta-Rev3		ATTTCTGCCAGCTGTTCAGC
ackA-Fwd	*ackA*	TTTCTGCTATCCGCAACG
ackA-Rev		TTGGTTGGGATAACCACCG
cyaA-Fwd	*cyaA*	CAATCAGGTGGTGCATTTCG
cyaA-Rev		AGAACTGGATGATCCCTTCG
flgJ-Fwd	*flgJ*	AACGCAGATCGATTTACGCC
flgJ-Rev		TCATCGTAGTTACGTGGCAC
gspE-Fwd	*gspE*	CATTCCTGCTTTCGTCTTCC
gspE-Rev		AAGCGTCAGTTCACTATGGC
cpxP-Fwd	*cpxP*	ATGATGCGCATAGTTACCGC
cpxP-Rev		ACTACTGGGAACGTGAGTTG
yhbO-Fwd	*yhbO*	GATCATGGGGATTAAACACC
yhbO-Rev		ACACCGGTTTGCCACTATTC
plsB-Fwd	*plsB*	GCACGTCAATGTGCTTTACC
plsB-Rev		TCTCTCTGATTACCCTTCGC
ydhK-Fwd	*ydhK*	ATGAACGCATCGTCATGGTC
ydhK-Rev		TGCCAGAGTAAACTGGCATG
entE-Fwd	*entE*	TATTTCCAGCTTTCCGGTGG
entE-Rev		AGTGGATTTCCTTCGGCATC
flhC-Fwd	*flhC*	GACGATTACTCAGTTGACGC
flhC-Rev		AACAAACCGCACCAATGTCC
ynbB-Fwd	*ynbB*	ATGGTGATTTCAGGTCCACG
ynbB-Rev		CCTTCCAATGTTTTTCCCGG
glnS-Fwd	*glnS*	TTACGCACTCTCTGTGTACG
glnS-Rev		AGCACCAGACGTTTGTACTG
yhjG-Fwd	*yhjG*	TATTCCCGAAGTCACGATGG
yhjG-Rev		AAAGCAACACGGGTGTTACC
ftsI-Fwd	*ftsI*	CGATTACCAAAGTTGACCCC
ftsI-Rev		ACAGCTACAAAGAGATCGCC
yhjX-Fwd	*yhjX*	AAACCAGCAATGGTGTGGTG
yhjX-Rev		AGTGAGGCGATAATCGAACC

Nucleotides written in lowercase were added for cloning purposes.

Table S2. Summary of suppressor mutants in terms of numbers of genes harboring mutation(s).

No. of genes with mutation(s) in each suppressor mutant	Days of isolation	No. of suppressor mutants	Suppressor clone
1	2	15	#4-2, #5-1, #8-2, #11-2, #12-2, #13-3, #16-2, #18-1, #19, #20, #21, #24, #25, #27, #29
	3	12	#31-1, #33-1, #35-1, #37-1, #39-2*, #40-2, #43-1, #44-1, #45-1, #46-1, #51, #57
	4	3<br>(total 30)	#64-1, #65-2, #70
2	2	5	#3, #10-1, #15-1, #17-2, #23
	3	3	#32-1, #48-1, #54
	4	1<br>(total 9)	#68-1
3	2	1	#14-1
	3	0	-
	4	0<br>(total 1)	-

* Suppressor mutant #39-2 harbors two distinct point mutations in *aceE*.

Table S3. Summary of suppressor mutants in terms of genes mutated.

Mutation occured	Mutation gene	No. of suppressor mutants	No. of suppressor mutants with one, two or three mutation gene(s)		
			one	two*1	three*2
Pta-AckA pathway	*pta*	17	14	3	-
	ackA	1	1	-	-
		(Total 18)			
PDH complex	*aceE*	11	5	5	1
	aceF	2	2	-	-
	lpd	3	3	-	-
		(Total 16)			
Others	*cyaA*	4	2	2	-
	entE	1	1	-	-
	ynbB	1	1	-	-
	yhjG	1	1	-	-
	gspE	1	-	1	-
	plsB	1	-	1	-
	ydkK	1	-	1	-
	flgJ	1	-	1	-
	cpxP	1	-	-	1
	yhbO	1	-	-	1
	yhiX	1	-	1	-
	flhC	1	-	1	-
	ftsI	1	-	1	-
	glnS	1	-	1	-

*1 Suppressor mutants #15-1, #17-2, and #23, which carry the *pta* mutation, concurrently harbor a second mutation in *gspE*, *plsB*, and *ydhK*, respectively. Suppressor mutants #3, #10-1, #32-1, #48-1, and #54, which carry the *aceE* mutation, concurrently harbor a second mutation in *flgJ*, *cyaA*, *yhiX*, *flhC*, and *ftsI*, respectively. Suppressor mutant #68-1 harbors mutations in *cyaA* and *glnS*.
*2 Suppressor mutant #14-1 carries three mutations, in *aceE*, *cpxP*, and *yhbO*.
